# Beyond morphology: cryptic diversity in 9 new *Gyrodactylus* species (Monogenea: Gyrodactylidae) associated with northwest African barbels (Cyprinidae) and remarks on *Gyrodactylus nyingiae* Shigoley, Rahmouni, Louizi, Pariselle and Vanhove, 2023

**DOI:** 10.1017/S0031182025000411

**Published:** 2025-04

**Authors:** Chahrazed Rahmouni, Mária Seifertová, Andrea Šimková

**Affiliations:** Department of Botany and Zoology, Faculty of Science, Masaryk University, Brno, Jihomoravský, Czech Republic

**Keywords:** cyprinids, gyrodactylids, monogenea, Morocco, Southwest Mediterranean

## Abstract

Cryptic diversity, characterized by morphologically similar but genetically distinct species, poses significant challenges to traditional taxonomic methods. Within monogeneans parasitizing northwest African barbels, this complexity hampers species identification, limiting our understanding of diversity, distribution and evolutionary relationships. Supported by previously published genetic data, we morphologically delineate herein 9 *Gyrodactylus* species from Morocco. Newly described species include *G. agnesei* sp. nov. and *G. benhoussai* sp. nov. from *Luciobarbus rabatensis*, with the latter also found on *Carasobarbus fritschii*, and both *G. deburonae* sp. nov. and *G. marruecosi* sp. nov. from *L. massaensis*. Additionally, *G. diakini* sp. nov. and *G. louiziae* sp. nov. were identified from *L. rifensis* and *L. yahyaouii*, respectively. *Pterocapoeta maroccana* harboured *G. pterocapoetai* sp. nov., morphologically resembling *G. shigoleyae* sp. nov. from sympatric *L. zayanensis*. We also examined taxonomical discrepancies between *Gyrodactylus* species from *L. ksibi* and *L. pallaryi*, evaluated the status of previously described *G. nyingiae* and described *G. qninbai* sp. nov. from *L. ksibi*. Our findings highlight the conservative morphology in northwest African *Gyrodactylus*, characterized by an ancestral median ridge in the ventral bar membrane, similar to that found in species from Eurasia. Subtle phenotypic features, like bifurcations in dorsal bars and proportions of marginal hooks, serve as diagnostic traits. We further evidenced a potential host-switching event from northwest African to Iberian hosts, correlating with the region’s geological history and cyprinid dispersal events during intermittent closures of the Strait of Gibraltar. These insights illuminate the complex evolutionary processes driving gyrodactylid diversification in the West Mediterranean.

## Graphical abstract

**Figure:**
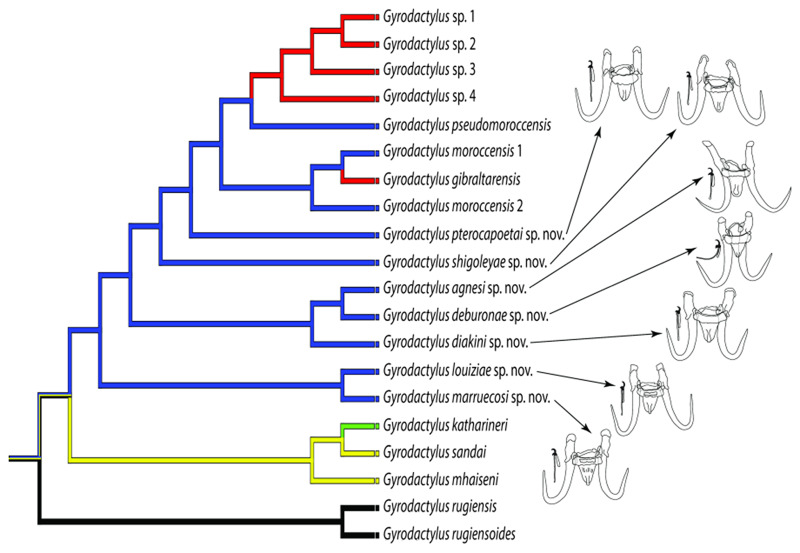

## Introduction

To date, the viviparous parasites of *Gyrodactylus* von Nordmann, 1832 (Gyrodactylidae van Beneden et Hesse, 1832) encompass over 600 valid species known to parasitize a wide range of teleost fish worldwide (Harris et al., 2004; Bakke et al., 2007; Pugachev et al., 2010). Previous surveys focused on the parasite fauna of North African cyprinid fish have revealed high species diversity in terms of *Dactylogyrus* (Dactylogyridae), with 17 species of the genus recorded in the region thus far (Řehulková et al., 2021). In contrast, among the 43 *Gyrodactylus* species reported in African freshwater ichthyofauna (Řehulková et al., 2018; Přikrylová et al., 2021, 2024), a single northwest African gyrodactylid species, *Gyrodactylus nyingiae* Shigoley, Rahmouni, Louizi, Pariselle and Vanhove, 2023, was described from *Luciobarbus pallaryi* (Pellegrin, 1919) and was also reported on *Luciobarbus ksibi* (Boulenger, 1905) (Shigoley et al., 2023). Subsequently, Rahmouni et al. (2023a) investigated ectoparasitic flatworms from native cyprinid hosts occurring in northwest Africa (Morocco) and Iberia (Portugal and Spain), providing formal descriptions of 3 *Gyrodactylus* species found on a range of endemic West Mediterranean cyprinids. These include *Gyrodactylus gibraltarensis* Rahmouni, 2023, from the Iberian *Luciobarbus graellsii* (Steindachner, 1866); *Gyrodactylus moroccensis* Rahmouni, 2023, from various northwest African *Luciobarbus* species (*Luciobarbus rabatensis* Doadrio, Perea and Yahyaoui, 2015 (type-host); *Luciobarbus yahyaouii* Doadrio, Casal-Lopez and Perea, 2016; *Luciobarbus maghrebensis* Doadrio, Perea and Yahyaoui, 2015; *Luciobarbus zayanensis* Doadrio, Casal-Lopez and Yahyaoui, 2016; and *Luciobarbus rifensis* Doadrio, Casal-Lopez and Yahyaoui, 2015); and *Gyrodactylus pseudomoroccensis* Rahmouni, 2023, from *Luciobarbus ksibi* (Boulenger, 1905). Notably, these species exhibited a distinct morphotype of haptoralsclerotized structures, characterized by an atypical T-shaped dorsal bar, a feature observed for the first time in *Gyrodactylus* species and seemingly restricted to the West Mediterranean region. Additionally, their haptoral morphology, along with that of 12 previously undescribed northwest African and Iberian *Gyrodactylus* species presented in Rahmouni et al. (2023a), resembled that of their European and Middle Eastern congeners, supporting historical dispersal trajectories of cyprinids from the Middle East, the centre of speciation for freshwater fauna to the West Mediterranean (Por, 1989). Molecular data further supported the relatedness between Eurasian and West Mediterranean *Gyrodactylus* lineages (Rahmouni et al., 2023a).

In this paper, we describe 9 *Gyrodactylus* species collected from endemic cyprinid hosts in Morocco. This study provides the first parasitological data on gyrodactylids hosted by 3 Moroccan cyprinids, *Carasobarbus fritschii* (Günther, 1874), *Luciobarbus massaensis* (Pellegrin, 1922) and *Pterocapoeta maroccana* (Günther, 1902) which had not been previously examined. Shigoley et al. (2023) previously reported the presence of *Gyrodactylus* species on *P. maroccana, C. fritschii* and *L. massaensis*; however, formal descriptions were not provided due to the limited number of monogenean specimens collected. We also discuss the taxonomic status of *G. nyingiae*, recently described from *L. ksibi* and *L. pallaryi* sampled from disparate locations across Morocco. Rahmouni et al. (2023a) already highlighted the possible misidentification of specimens collected from *L. ksibi*, referred to as *G. nyingiae*, as their haptoral sclerites notably differed from those collected from *L. pallaryi*. Additionally, the noticeable and unusual range in the size of the haptoral sclerites of *G. nyingiae* from *L. pallaryi* reported by Shigoley et al. (2023) is uncommon among *Gyrodactylus* specimens parasitizing a single fish population. Finally, we retrieved the phylogenetic tree previously published in Rahmouni et al. (2023a) to construct a mapping of the geographical distribution to explore the origin and pattern of host switching within the West Mediterranean *Gyrodactylus* lineage.

## Material and methods

### Fish and parasite sampling and identification

The parasitological survey, conducted in 2015, involved the collection of a total of 128 fish specimens comprising 9 cyprinid species from various Moroccan watersheds. Fish specimens were identified on-site by local collaborators (listed in the acknowledgements). Upon transporting the fish to the laboratory, the external body surfaces, fins and gills of cyprinid hosts were examined for the presence of *Gyrodactylus* using an MST130 stereoscopic microscope. Specimens were carefully extracted from skin and gills using surgical needles. A selected number was entirely fixed and mounted on slides with a mixture of glycerine and ammonium picrate (GAP), following the method described by Malmberg (1957). Another selected number was bisected, with the haptoral part mounted on slides and the anterior part preserved in ethanol for genetic studies, as detailed in Rahmouni et al. (2023a). The terminology for haptoral sclerites and the method of measurement followed the guidelines provided by Malmberg (1970) and Pugachev et al. (2010). Measurements of specimens were obtained using an Olympus BX51 phase-contrast microscope and Olympus Stream Image Analysis software version 1.9.3 (Olympus, Tokyo, Japan) and are reported in micrometres as the mean followed by the range and the number of specimens measured (*n*) in parentheses. Illustrations of the haptoral sclerotized parts were produced from flattened specimens using an Olympus BX51 microscope equipped with a drawing tube and subsequently edited using Adobe Illustrator CS6 version 16.0.0 and Adobe Photoshop version 13.0 (Adobe Systems Inc., San Jose, CA, USA). Infection indices (prevalence (P) and intensity of infection (I)) were calculated following Bush et al. (1997). The type-material (in GAP) was deposited in the Helminthological Collection of the Institute of Parasitology, Biology Centre of the Academy of Sciences of the Czech Republic, in České Budějovice (IPCAS) under the accession numbers (M–804-12). Prior to this research, we genetically confirmed the conspecificity of the collected *Gyrodactylus* specimens from Morocco by amplifying and sequencing the 18S rDNA and the ITS regions (detailed protocols for the genetic part can be found in our previous publication in Rahmouni et al. 2023a). We then performed phylogenetic analyses that included sequences from the northwest African *Gyrodactylus* communities, along with a range of *Gyrodactylus* congeners from around the world (see Rahmouni et al. 2023a).

### Mapping of the geographical origin of West Mediterranean Gyrodactylus

To explore the geographical origin of *Gyrodactylus* lineage, particularly focusing on the West Mediterranean region, we utilized the phylogenetic tree generated in our previous study (Rahmouni et al., 2023a) using Maximum Likelihood (ML) and Bayesian Inference (BI) methods. Combined ML and BI trees were pruned using TreeGraph v. 2.15 (Müller and Müller, 2004) to select Clade A, which comprised *Gyrodactylus* species parasitizing cyprinid fish found across the Strait of Gibraltar, particularly in northwest Africa and Iberia, and 2 Middle Eastern *Gyrodactylus* species and 1 European *Gyrodactylus* species. Clade A encompassed 18 species in total, including the species that were unknown at that time (*Gyrodactylus* sp. 5–11) and which we describe below, while Clade B, retained as the most basal clade to Clade A, contained 2 European species. The mapping of geographical distribution onto the pruned phylogenetic tree was carried out using Mesquite v3.61 (Maddison and Maddison, 2019, http://mesquiteproject.org). This process involved annotating each species with its corresponding geographical distribution (Iberia, northwestern Africa and Eurasia), which was mapped onto the phylogenetic tree. The final tree was manually edited using Photoshop v13.0.

## Results

The Life Science Identifier (LSID) for this publication is: urn: lsid:zoobank.org:pub:4836137B-4F95-47D1-BD24-B42CFD4E8A B4.

### Gyrodactylus agnesei sp. nov. (Figure 1A)

**Zoobank registration**: urn:lsid:zoobank.org:act:53A95698-FF15-4AED-BF12-A3F932CEDB6F

**Synonym**: *Gyrodactylus* sp. 7 *sensu* Rahmouni et al. (2023a) (18S: OR807830, ITS: OR807841)

**Type–host**: *Luciobarbus rabatensis* Doadrio, Perea and Yahyaoui, 2015 (Cyprinidae)

**Type–locality**: Maleh River (33°31’58.00” N, 06°37’39.06” W), Morocco

**Additional locality**: Grou River, Morocco

**Type–material**: 1 holotype and 2 paratypes (IPCAS, M–812)

**Site on the host**: Gill filaments

**Infection indices**: 11 specimens studied, *P* = 27%, *I* = 1–17 parasite specimens per infected host.

**Etymology**: The specific epithet ‘*agnesei*’ of the type-species is derived from the name of Dr Jean-François Agnèse, in recognition of his significant contributions to the field of fish parasitology in the African continent, during his tenure at Montpellier University (Montpellier, France).

#### Description

Based on 7 coverslip-flattened specimens. Haptor distinct fromrest of the body. Slender hamuli with well-developed, narrow, and outwardly curved inner roots. Tips of inner roots with hat-like cover, and folds typically observed in posterior part of base appear absent. Hamuli total length 69.4 (68.3–71.1), inner root length 26.1 (23.9–28.5), shaft length 49.5 (47.7–50.8) and point length 25.3 (24.1–26.4). Ventral bar 27 (25.9–28.4) long, 23.9 (22.5–25.2) wide, median width 5.4 (5–6), with blunt, short bilateral processes that extend outward from bar 2.3 (1.8–2.6) long, distance between lateral processes 23.9 (21–26.1) long, shield-shaped membrane approximately one-third length of hamuli shaft, blunt posteriorly with median ridge, 18.8 (15.8–21.5) long, 15.4 (13.4–16.5) wide. Dorsal bar irregular, attenuated extremities inserted into terminal plates, 23.4 (20.4–26.6) long, 2.2 (1.6–3.6) wide at midpoint. Marginal hooks total length 29.4 (27.6–31.1), handle length 22.9 (21.7–24.7), filament loop length 11.3 (8.7–13.1), sickle length to shaft attachment 6.7 (6.5–7.3), shaft length of sickle 5 (2.2–6.8), sickle distal width 6.6 (5.9–7.2), sickle proximal width 4.7 (4.3–5), point length of sickle 3 (2.1–3.6). Sickle foot with inconspicuous shelf and rounded heel. Sickle proper convex, gradually curved forward from base int straight, far-reaching point positioned above edge of prominent finger-like sickle toe. Male copulatory organ (MCO) spherical, 14.5 (13.3–16.3) in length and 14.4 (13.9–15.3) in width, armed with single apical spine and row of 8 spinelets, with bilateral pair of larger-sized spinelets.

#### Remarks

**Figure 1.**
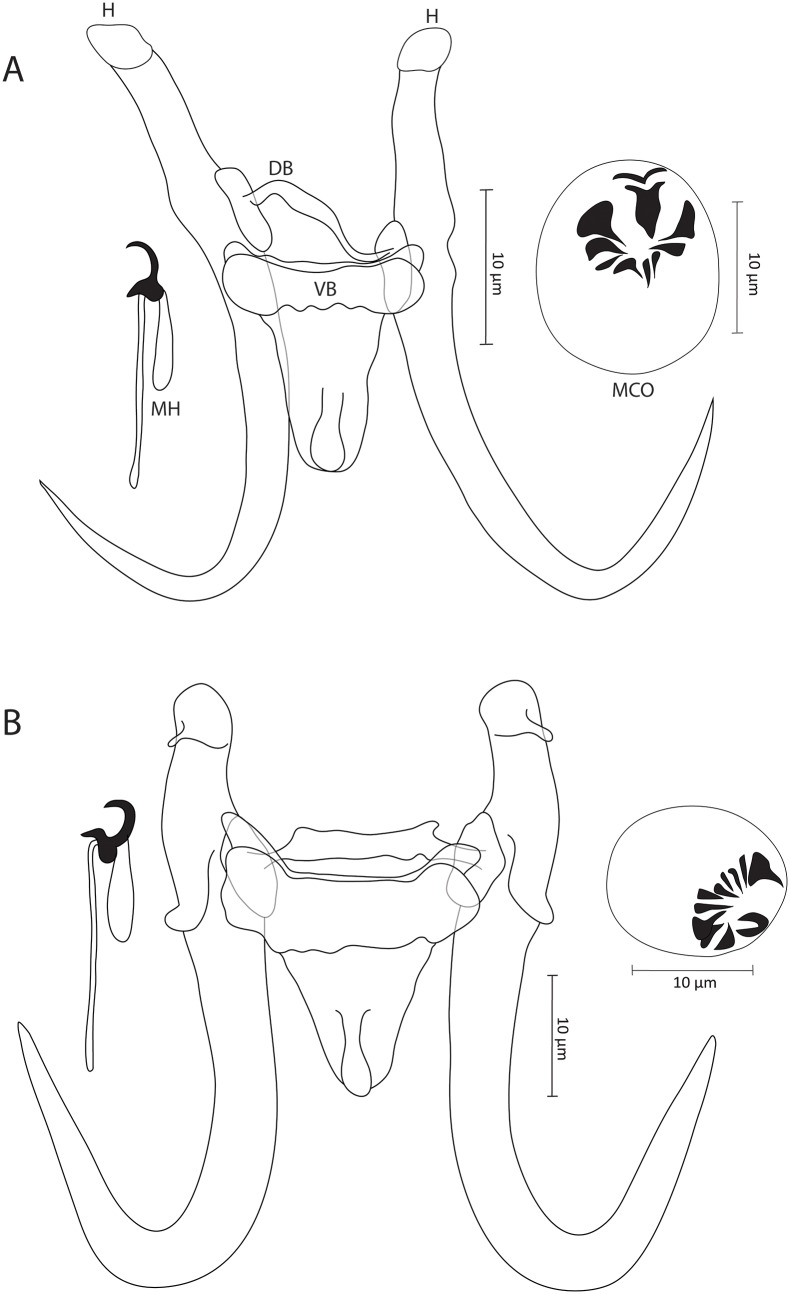
Illustrations of the sclerotized parts of (A) *Gyrodactylus agnesei* sp. Nov. From *Luciobarbus rabatensis* Doadrio, Perea and Yahyaoui, 2015 and (B) *Gyrodactylus benhoussai* sp. nov. from *Luciobarbus rabatensis* Doadrio, Perea and Yahyaoui, 2015. Abbreviations: DB, dorsal bar; H, hamuli; MCO, male copulatory organ; MH, marginal hooks; VB, ventral bar.

*Gyrodactylus agnesei* sp. nov. (Figure 1A) is the second gyrodactylid species identified to parasitize *L. rabatensis*, an endemic cyprinid of northwest Africa. It is differentiated from its congener *G. moroccensis*, previously described from the same host, by the absence of the accessory anterior portion of the hamuli and dorsal bar with a common shape (not T-shaped), features which are lacking in *G. moroccensis*, in addition to the T-shaped dorsal bar observed in *G. moroccensis* (Rahmouni et al., 2023a). *Gyrodactylus agnesei* sp. nov. exhibits similarities with *G. nyingiae* from *L. pallaryi*, another inhabitant of the same area, particularly in terms of the haptoral morphotype, characterized by slender hamuli and a ventral bar with short bilateral processes. It is worth noting that measurements of *G. nyingiae* were conducted using conserved specimens in ethanol, which were subsequently fixed in Hoyer’s medium, whereas our specimens were freshly fixed *in situ* in GAP. Fankoua et al. (2017) previously highlighted variability in the size of the haptoral sclerites of monogeneans associated with these 2 mounting procedures, with Hoyer’s medium resulting in significantly larger structures. In comparison to *G. nyingiae*, the new species *G. agnesei* sp. nov. exhibits shorter marginal hooks (27.6–31.1 μm in *G. agnesei* sp. nov. vs. 31.7–42.1 μm in *G. nyingiae*) and associated structures, namely, a shorter handle (21.7–24.7 μm in *G. agnesei* sp. nov. vs. 26.1–33.4 μm in *G. nyingiae*) and a longer filament (8.7–13.1 μm in *G. agnesei* sp. nov. vs. 5–5.9 μm in *G. nyingiae*) (the terms ‘shaft’ and ‘aperture’ were used for the handle and filament loop, respectively, in Shigoley et al. 2023). Additionally, the median ridge observed in the posterior edge of the ventral bar in *G. agnesei* sp. nov., as well as in all previously described species from Morocco (as discussed below), was not mentioned for *G. nyingiae*. This feature appears visible in the micrographs embedded by Shigoley et al. (2023), leading us to believe that the authors may have overlooked it (see the drawing of the ventral bar of *G. nyingiae*). Nonetheless, the most distinctive feature separating these 2 species is the shape of the hamuli. In *G. agnesei* sp. nov., this structure is slender, with well-developed, narrow and outwardly curved inner roots. In contrast, the hamuli of *G. nyingiae* exhibit either twisted extremities or robust and shorter inner roots.

### Gyrodactylus benhoussai sp. nov. (Figure 1B)

**Zoobank registration**: urn:lsid:zoobank.org:act:B143338B-BE72-4684-8A61-DD49B08 F49EE

**Type–host**: *Luciobarbus rabatensis* Doadrio, Perea and Yahyaoui, 2015 (Cyprinidae)

**Additional host**: *Carasobarbus fritschii* (Günther, 1874) (Cyprinidae) from the same locality

**Type–locality**: Maleh River (33°31’58.00” N, 06°37’39.06” W) for both hosts, Morocco

**Type–material**: 1 holotype and 2 paratypes (IPCAS, M–809)

**Site on the host**: Gill filaments for both hosts

**Infection indices**: 11 specimens of *L. rabatensis* and 41 specimens of *C. fritschii* were studied, *P* = 9.09%, *I* = 1–2 for *L. rabatensis*; *P* = 9.75%, *I* = 1–2 for *C. fritschii*.

**Etymology**: The epithet ‘*benhoussai*’ of the type-species is dedicated to Prof. Abdelaziz Benhoussa from Mohamed V University (Rabat, Morocco), acknowledging his significant contributions to fish parasitology research. Prof. Benhoussa generously facilitated field trips and fish sampling in 2015, demonstrating his commitment to advancing scientific knowledge in this field.

#### Description

Based on 5 coverslip-flattened specimens from *L. rabatensis*, and 2 specimens from *C. fritschii*. Haptor distinct from rest of the body. Slender hamuli, with well-developed, narrow and inwardly curved inner roots. Tips of inner roots with hat-like cover, with folds present in posterior part of base. Hamuli total length 61.4 (59.1–66), inner root length 19.5 (17.6–22.5), shaft length 44.8 (43–48.1) and point length 30.4 (27.9–32.1). Ventral bar 27 (25–29.5) long, 27.3 (24.6–29.7) wide, median width 7.4 (6.2–8.2), with blunt and short bilateral processes that extend outward from bar 2.8 (2–3.6) long, distance between lateral processes 27.4 (24.7–29.3) long, shield-shaped membrane, blunt posteriorly with median ridge, 16.1 (15.4–17) long, 15.1 (14.3–16.6) wide. Dorsal bar simple and straight with attenuated extremities inserted into terminal plates, 27.6 (26.4–31.8) long, 2.4 (1.5–2.9) wide at midpoint. Marginal hooks total length 29.7 (27.1–33.5), handle length 23.7 (21.7–27.2), filament loop length 10.4 (9.1–11.8), sickle length to shaft attachment 5.7 (5.2–6.4), shaft length of sickle 4.4 (3.7–5), sickle distal width 4.8 (4.2–5.5), sickle proximal width 3.7 (2.8–4.5), point length of sickle 1.9 (1.6–2.5). Sickle foot with conspicuous shelf and flat, slightly rounded heel. Sickle proper convex and gradually curved forward from base into downward point positioned above edge of prominent finger-like sickle toe. MCO spherical, 14.8 (11.2–18.4) in length and 13.8 (12.3–15.2) in width, armed with single apical spine and row of 10 spinelets, with bilateral pair of larger-sized spinelets.

#### Remarks

Thus far, *L. rabatensis* has only been reported as a host for a few gyrodactylid specimens (Rahmouni et al., 2023a and this study), with one of them also found on *C. fritschii* sampled from the same watershed. At the host species level, no intraspecific variation in the sclerotized structures of *G. benhoussai* sp. nov. was observed. *Gyrodactylus benhoussai* sp. nov. (Figure 1B) can be readily distinguished from *G. moroccensis*, recently described from the same host (Rahmouni et al., 2023a), by the absence of the T-shaped dorsal bar and the accessory membranous structure associated with the hamuli, which are present in *G. moroccensis*. When compared to *G. agnesei* sp. nov., also found on *L. rabatensis, G. benhoussai* sp. nov. exhibits different characteristics of its hamuli, notably: (i) slightly shorter inner roots (17.6–22.5 μm in *G. benhoussai* sp. nov. vs. 23.9–28.5 μm in *G. agnesei* sp. nov.) with a cover-like structure that diminishes posteriorly, (ii) more sturdy shafts, (iii) longer points (27.9–32.1 μm in *G. benhoussai* sp. nov. vs. 24.1–26.4 μm in *G. agnesei* sp. nov.).

### Gyrodactylus deburonae sp. nov. (Figure 2A)

**Zoobank registration**: urn:lsid:zoobank.org:act:53590D5E-3A1E -4156-9537-1059EB484A02

**Synonym**: *Gyrodactylus* sp. 8 *sensu* Rahmouni et al. (2023a) (18S: OR807831, ITS: OR807842)

**Type–host**: *Luciobarbus massaensis* (Pellegrin, 1922) (Cyprinidae)

**Type–locality**: Tamrhakht (30°31’33.06” N, 09°38’53.06” W), Morocco

**Type–material**: 1 holotype and 2 paratypes (IPCAS, M–805)

**Site on the host**: Fins

**Infection indices**: 11 specimens studied, *P* = 75%, *I* = 1–2 parasite specimens per infected host.

**Etymology**: The specific epithet ‘*deburonae*’ of the type-species is dedicated to Prof. Isaure de Buron from the College of Charleston (Charleston, USA), in acknowledgement of her substantial contributions to the fields of ecology and evolutionary fish parasitology through her extensive research efforts.

#### Description

Based on 3 coverslip-flattened specimens. Haptor distinct from rest of the body. Slender hamuli with well-developed and inwardly curved inner roots. Tips have hat-like cover, with folds present in posterior part of base. Hamuli total length 46.6 (46.4–46.8), inner root length 14.1 (10.8–16), shaft length 36.4 (36.2–36.7) and point length 20.9 (20.7–21.2). Ventral bar 17.1 (15.5–18.4) long, 16.6 (16.2–17.3) wide, median width 4.5 (3.9–4.9), with blunt and short bilateral processes that extend outward from bar 1.9 (1.6–2.2) long, distance between lateral processes 16.3 (15.8–16.7) long, shield-shaped membrane, blunt posteriorly with median ridge, 8.6 (7.9–10) long, 10.3 (9.3–11.4) wide. Dorsal bar simple and curved with attenuated extremities inserted into terminal plates, 17.2 (15.9–18.7) long, 1.9 (1.4–2.7) wide at midpoint. Marginal hooks total length 22.8 (21.9–23.5), handle length 18.3 (16.4–19.4), filament loop length 10.1 (9.1–11.2), sickle length to shaft attachment 7.1 (5.1–8.9), shaft length of sickle 4.5 (5.2–4.9), sickle distal width 5 (4.7–5.1), sickle proximal width 3.4 (3.2–3.6), point length of sickle 1.4 (1.3–1.4). Sickle foot with conspicuous shelf and rounded heel. Sickle proper convex and gradually curved forward from base into straight point positioned above edge of prominent finger-like sickle toe. MCO not observed.

#### Remarks

**Figure 2.**
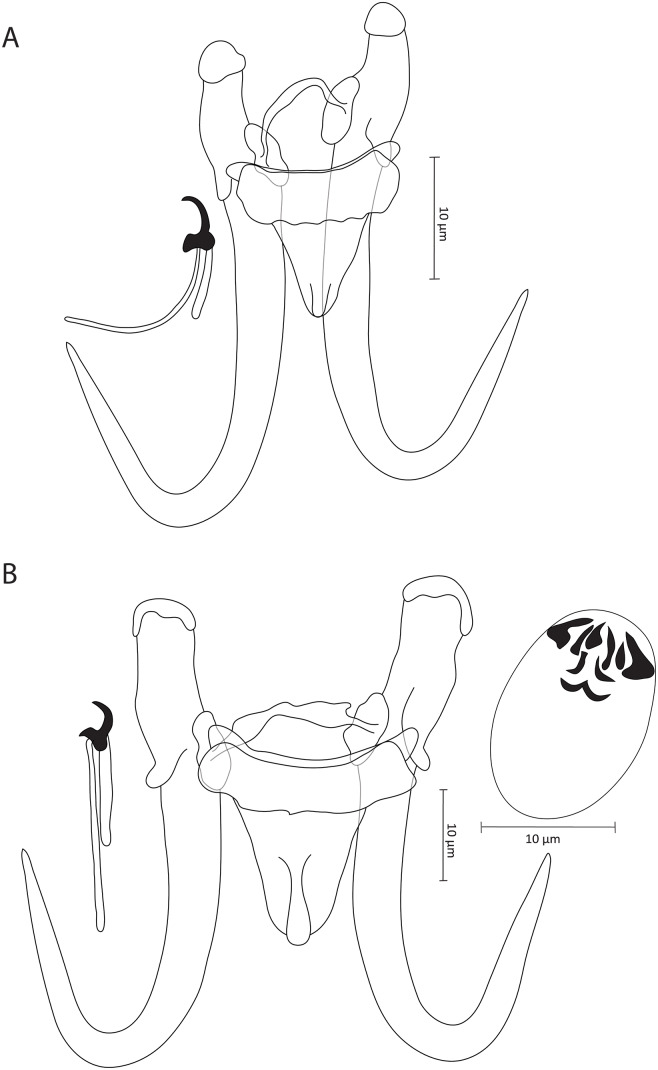
Illustrations of the sclerotized parts of (A) *Gyrodactylus deburonae* sp. nov. from *Luciobarbus massaensis* (Pellegrin, 1922) and (B) *Gyrodactylus diakini* sp. nov. from *Luciobarbus rifensis* Doadrio, Casal-lópez and Perea 2015.

This represents the second recorded instance of a gyrodactylid species on northwest African *L. massaensis*. Previously, Shigoley et al. (2023) encountered a single specimen, which unfortunately did not allow for a formal description or measurement of the hard parts. Thus, the morphology of the sclerotized structures and meristic data of a gyrodactylid from *L. massaensis* are presented herein for the first time. In light of the previous study by Rahmouni et al. (2023a), the newly described *G. deburonae* sp. nov. (Figure 2A) is primarily differentiated from its genetically closely related congener *G. agnesei* sp. nov., as described above, by having (i) shorter hamuli (46.4–46.8 μm in *G. deburonae* sp. nov. vs. 68.3–71.1 μm in *G. agnesei* sp. nov.), with inner roots curved in opposite directions, (ii) shorter ventral bars (15.5–18.4 μm in *G. deburonae* sp. nov. vs. 25.9–28.4 μm in *G. agnesei* sp. nov.) and (iii) shorter dorsal bars (15.9–18.7 μm in *G. deburonae* sp. nov. vs. 20.4–26.6 μm in *G. agnesei* sp. nov.).

### Gyrodactylus diakini sp. nov. (Figure 2B)

**Zoobank registration**: urn:lsid:zoobank.org:act:CD85451C-49B0 -4EE6-A3A7-E9522B359CF7

**Synonym**: *Gyrodactylus* sp. 9 *sensu* Rahmouni et al. (2023a) (18S: OR807832, ITS: OR807843)

**Type–host**: *Luciobarbus rifensis* Doadrio, Casal-Lopez and Yahyaoui, 2015 (Cyprinidae)

**Type–locality**: Tributary of Loukkos (34°54’57.02” N, 05°32’17.02” W), Morocco

**Type–material**: 1 holotype and 2 paratypes (IPCAS, M–811)

**Site on the host**: Fins

**Infection indices**: 10 specimens studied, *P* = 50%, *I* = 1–2 parasite specimens per infected host.

**Etymology**: The epithet ‘*diakini*’ of the type-species is given to honour the memory of Dr Andrei Diakin, a colleague from Masaryk University (Brno, Czech Republic), who conducted research on parasitic protozoans.

#### Description

Based on 6 coverslip-flattened specimens. Haptor distinct from rest of the body. Stout hamuli, with well-developed and straight inner roots. Tips have hat-like cover, with folds present in posterior part of base. Hamuli total length 62.9 (61.3–65.9), inner root length 21 (20.4–21.7), shaft length 45 (43.4–46.2) and point length 29.5 (27.9–31.9). Ventral bar 28.5 (27.5–29) long, 26.4 (25.4–27.1) wide, median 7.4 (6.8–8), with blunt and short bilateral processes that extend outward from bar 2.3 (2.1–2.5) long, distance between lateral processes 25.9 (24.7–26.5) long, shield-shaped membrane, blunt posteriorly with median ridge, 8.6 (7.9–10) long, 10.3 (9.3–11.4) wide. Dorsal bar curved at midpoint, with posterior projections near attenuated extremities inserted into terminal plates, 27.8 (26.8–28.4) long, 2.3 (1.9–2.6) wide at midpoint. Marginal hooks total length 28.6 (27.6–29.8), handle length 23 (22.3–23.8), filament loop length 10.5 (9.1–11.7), sickle length to shaft attachment 5.4 (5.1–5.6), shaft length of sickle 4.7 (4.2–5), sickle distal width 5.1 (4.7–5.4), sickle proximal width 3.5 (3.4–3.6), point length of sickle 1.6 (1–1.8). Sickle foot with conspicuous shelf and globous heel. Sickle proper curved distally, point straight exceeding edge of downward finger-like sickle toe. MCO oval, 14.8 (11.2–18.4) in length and 13.8 (12.3–15.2) in width, armed with single apical spine and row of 8 spinelets, with bilateral pair of larger-sized spinelets.

#### Remarks

*Gyrodactylus diakini* sp. nov. (Figure 2B) is the second species identified on *L. rifensis* from northwest Africa (Morocco) (Rahmouni et al., 2023a). It can be easily distinguished from *G. moroccensis*, found on the same host, by its typical haptoral structures, unlike *G. moroccensis*, known for its characteristic accessories of the hamuli and T-shaped dorsal bar. According to Rahmouni et al. (2023a), *G. diakini* sp. nov. is genetically closely related to *G. agnesei* sp. nov. and *G. deburonae* sp. nov., with a divergence exceeding the limit value (5% and 7.4%, respectively) (Ziȩtara and Lumme, 2003; Huyse et al., 2004; Rahmouni et al., 2023b). Morphologically, *G. diakini* sp. nov. possesses larger haptoral structures compared to those of *G. deburonae* sp. nov. The main differences lie in (i) the longer hamuli (61.3–65.9 μm in *G. diakini* sp. nov. vs. 46.4–46.8 μm in *G. deburonae* sp. nov.), (ii) the longer ventral bar (27.5–29 μm in *G. diakini* sp. nov. vs. 15.5–18.4 μm in *G. deburonae* sp. nov.) and finally (iii) the longer dorsal bar (26.8–28.4 μm in *G. diakini* sp. nov. vs. 15.9–18.7 μm in *G. deburonae* sp. nov.). *Gyrodactylus diakini* sp. nov. exhibits smaller hamuli than those of *G. agnesei* sp. nov. (61.3–65.9 μm in *G. diakini* sp. nov. vs. 68.3–71.1 μm in *G. agnesei* sp. nov.). It should be noted that the apical spine and the single row of spinelets of the MCO of *G. diakini* sp. nov. that were visualized may not be in their natural position due to the fixation procedure, which most likely shifted the spines.

### Gyrodactylus louiziae sp. nov. (Figure 3A)

**Zoobank registration**: urn:lsid:zoobank.org:act:7CB55CBB-075B -40B9-B950-B99FB0EB2A8F

**Synonym**: *Gyrodactylus* sp. 10 *sensu* Rahmouni et al. (2023a) (18S: OR807833, ITS: OR807844)

**Type–host**: *Luciobarbus yahyaouii* Doadrio, Casal-Lopez and Perea, 2016 (Pellegrin, 1924) (Cyprinidae)

**Type–locality**: Meloulou River (34°10’51.07” N, 03°31’59.06” W), Morocco

**Type–material**: 1 holotype and 2 paratypes (IPCAS, M–808)

**Site on the host**: Fins

**Infection indices**: 14 specimens studied, *P* = 60%, *I* = 1–2 parasite specimens per infected host.

**Etymology**: The specific epithet ‘*louiziae*’ of the type-species is named in honour of Dr Halima Louizi from Mohamed V University (Rabat, Morocco), in recognition of her significant contributions to North African fish parasitology.

#### Description

Based on 4 coverslip-flattened specimens. Haptor distinct from the rest of the body. Stout hamuli with well-developed and inwardly curved inner roots. Tips have hat-like cover, with folds present in posterior part of base. Hamuli total length 61.8 (59.4–63.4), inner root length 18.8 (18.3–19.7), shaft length 46.7 (44.6–48.6) and point length 29.1 (28.3–30.7). Ventral bar 25.8 (24.4–27.1) long, 25.6 (24.8–26.4) wide, median 6.8 (5.7–7.6), with blunt and short bilateral processes that extend outward from bar 2.5 (2.3–2.7) long, distance between lateral processes 25.2 (23.5–27.3) long, shield-shaped membrane, blunt posteriorly with median ridge, 14.5 (14.1–15.1) long, 15.1 (14.1–16.5) wide. Dorsal bar straight, with posterior projections near attenuated extremities inserted into terminal plates, 27.7 (25.3–29.7) long, 2.2 (1.7–3) wide at midpoint. Marginal hooks total length 27.2 (25.1–28.6), handle length 20.8 (18.2–22.7), filament loop length 10.7 (10.2–11.4), sickle length to shaft attachment 5.8 (5.4–6.3), shaft length of sickle 4.3 (3.7–4.6), sickle distal width 5.2 (4.7–5.6), sickle proximal width 3.6 (2.4–4), point length of sickle 1.9 (1.4–2.8). Sickle foot with inconspicuous shelf and globous downward heel. Sickle proper curved distally with far-reaching point almost above edge of downward finger-like sickle toe. MCO oval, 14.8 (11.2–18.4) in length and 13.8 (12.3–15.2) in width, armed with single apical spine and row of 6 spinelets, with bilateral pair of larger-sized spinelets.

#### Remarks

**Figure 3.**
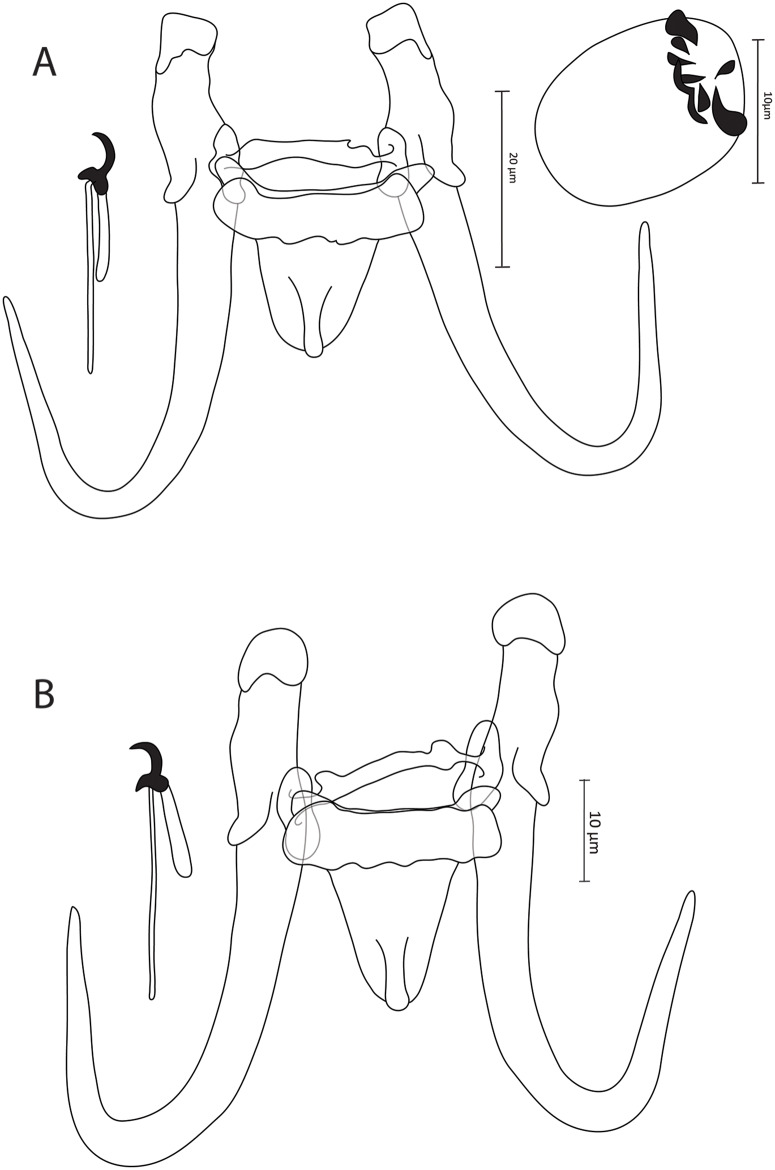
Illustrations of the sclerotized parts of (A) *Gyrodactylus louiziae* sp. nov. from *Luciobarbus yahyaouii* Doadrio, Casal-lopez and Perea, 2016 and (B) *Gyrodactylus marruecosi* sp. nov. from *Luciobarbus massaensis* (Pellegrin, 1922).

*Gyrodactylus louiziae* sp. nov. (Figure 3A) is the second species recognized on its host *L. yahyaouii* after the morphologically distinctive *G. moroccensis* (Rahmouni et al., 2023a). Meristic data of *G. louiziae* sp. nov. from *L. yahyaouii* and *G. diakini* sp. nov. from *L. rifensis* overlap significantly, and the morphotype of their haptoral sclerites are similar. However, these 2 species were previously discriminated genetically (Rahmouni et al., 2023a). Morphologically, *G. louiziae* sp. nov. and *G. diakini* sp. nov. can be distinguished from each other based on their (i) hamuli roots (curved inward in *G. louiziae* sp. nov. while straight in *G. diakini* sp. nov.). It should be noted that the apical spine and single row of spinelets of the MCO of *G. louiziae* sp. nov. that could be visualized were not in their natural position due to the fixation procedure, which likely shifted the garniture (spines) of the MCO.

### Gyrodactylus marruecosi sp. nov. (Figure 3B)

**Zoobank registration**: urn:lsid:zoobank.org:act:6B810A64-6E86-4324-BDC9-BA635D4960A5

**Synonym**: *Gyrodactylus* sp. 11 *sensu* Rahmouni et al. (2023a) (18S: OR807834, ITS: OR807845)

**Type–host**: *Luciobarbus massaensis* (Pellegrin, 1922) (Cyprinidae)

**Type–locality**: Tamrhakht (30°31’33.06” N, 09°38’53.06” W), Morocco

**Type–material**: 1 holotype and 2 paratypes (IPCAS, M–806)

**Site on the host**: Fins

**Infection indices**: 11 specimens studied, *P* = 45%, *I* = 1–2 parasite specimens per infected host.

**Etymology**: The specific epithet ‘*marruecosi*’ of the type-species is derived from Marruecos, the old Spanish name for Morocco, the country of origin of the species.

#### Description

Based on 3 coverslip-flattened specimens. Haptor distinct from the rest of the body. Stout hamuli with well-developed and straight inner roots. Tips have hat-like cover, with folds present in posterior part of base. Hamuli total length 63.5 (62.7–64.4), inner root length 19.1 (17.8–20.5), shaft length 46.7 (45.3–48.2) and point length 29.1 (27.2–30.5). Ventral bar 26.5 (25.1–28.6) long, 26.4 (26.1–26.7) wide, median 6.5 (5.9–7), with blunt and short bilateral processes that extend outward from bar 2.2 (1.5–2.7) long, distance between lateral processes 25 (24.2–26) long, shield-shaped membrane, blunt posteriorly with median ridge, 17.7 (16.9–18.7) long, 15.6 (15.2–16.2) wide. Dorsal bar straight, with posterior projections near attenuated extremities inserted into terminal plates, 27.7 (26.7–28.7) long, 2.6 (2.4–3) wide at midpoint. Marginal hooks total length 28 (27.8–28.1), handle length 21.1 (20.8–21.6), filament loop length 9.2 (8.6–9.7), sickle length to shaft attachment 5.2 (4.3–5.8), shaft length of sickle 4.5 (4.4–4.5), sickle distal width 4.6 (4.2–5.1), sickle proximal width 3.9 (3.5–4.1), point length of sickle 1.9 (1.2–2.3). Sickle foot with conspicuous shelf and globous downward heel. Sickle proper curved distally with straight point positioned above edge of downward finger-like sickle toe. MCO not observed.

#### Remarks

*Gyrodactylus marruecosi* sp. nov. (Figure 3B) is the second species recognized on *L. massaensis* after *G. deburonae* sp. nov. In addition to the genetic divergence above the limit value (Ziȩtara and Lumme, 2003; Huyse et al., 2004; Rahmouni et al., 2023b) previously recovered between these 2 species (10.3%, see Rahmouni et al. 2023a), they are easily distinguishable from each other by *G. marruecosi* sp. nov. having (i) longer hamuli (62.7–64.4 μm in *G. marruecosi* sp. nov. vs. 46.4–46.8 μm in *G. deburonae* sp. nov.), (ii) longer ventral bars (25.1–28.6 μm in *G. marruecosi* sp. nov. vs. 15.5–18.4 μm in *G. deburonae* sp. nov.), (iii) longer dorsal bars (26.7–28.7 μm in *G. marruecosi* sp. nov. vs. 15.9–18.7 μm in *G. deburonae* sp. nov.) and finally (iv) longer marginal hooks (27.8–28.1 μm in *G. marruecosi* sp. nov. vs. 21.9–23.5 μm in *G. deburonae* sp. nov.). Genetically, *G. louiziae* sp. nov. found on *L. yahyaouii* was the closest relative to *G. marruecosi* sp. nov. described herein from *L. massaensis* (Rahmouni et al., 2023a). Despite the overlap in their meristic data, these species are morphologically discriminated by having (i) differently shaped dorsal bars (those of *G. marruecosi* sp. nov. exhibiting prominent posterior projections, unlike those of *G. louiziae* sp. nov.), and (ii) differently shaped marginal hooks (*G. marruecosi* sp. nov. exhibiting a sickle proper that is comparatively point heading downward and well-curved, while that of *G. louiziae* sp. nov. appears thin with a far-reaching point).

### Gyrodactylus pterocapoetai sp. nov. (Figure 4A)

**Zoobank registration**: urn:lsid:zoobank.org:act:B28791B3-507D-4895-BF1C-676C0B979E30

**Synonym**: *Gyrodactylus* sp. 5 *sensu* Rahmouni et al. (2023a) (18S: OR807828, ITS: OR807839)

**Type–host**: *Pterocapoeta maroccana* (Günther, 1902) (Cyprinidae)

**Type–locality**: Oum Er-Rbia River near El Borj (33°00”58.07” N, 05°37”48.06” W), Morocco

**Type–material**: 1 holotype and 2 paratypes (IPCAS, M–810)

**Site on the host**: Gill filaments

**Infection indices**: 3 specimens studied, *P* = 33%, *I* = 1–6 parasite specimens per infected host.

**Etymology**: The specific epithet ‘*pterocapoetai*’ of the type-species refers to the type-host *P. maroccana* found only in Morocco.

#### Description

Based on 8 coverslip-flattened specimens. Haptor distinct from the rest of the body. Stout hamuli with well-developed and straight inner roots. Tips have hat-like cover, with folds present in posterior part of base. Hamuli total length 61.3 (58.1–63.1), inner root length 19.6 (18.9–20.8), shaft length 45.3 (43.9–46.9) and point length 28.4 (26.1–30.4). Ventral bar 23.3 (21.9–24.4) long, 23.3 (22.6–24) wide, median 7.3 (6.6–8.1), with blunt and short bilateral processes that extend outward from bar 2.9 (1.7–3.5) long, distance between lateral processes 23.3 (22.4–24.1) long, shield-shaped membrane, blunt posteriorly with median ridge, 13 (10.9–15.6) long, 13.4 (11.6–14.1) wide. Dorsal bar curved, posterior projections near attenuated extremities show bifurcations, 27 (25.7–28.6) long, 2.1 (1.8–2.5) wide at midpoint. Marginal hooks total length 32.1 (31.5–32.8), handle length 27.2 (26.4–27.8), filament loop length 11.4 (10.5–12), sickle length to shaft attachment 5.3 (5–5.8), shaft length of sickle 4.5 (4.3–4.7), sickle distal width 4.7 (4.3–5.1), sickle proximal width 3.6 (3.2–4.3), point length of sickle 1.6 (1.2–1.9). Sickle foot with conspicuous shelf and globous downward heel. Sickle proper curved distally with straight point positioned above sickle toe. MCO oval, 14.8 (11.2–18.4) in length and 13.8 (12.3–15.2) in width, armed with single apical spine and row of at least 7 spinelets; bilateral pair seems larger.

#### Remarks

**Figure 4.**
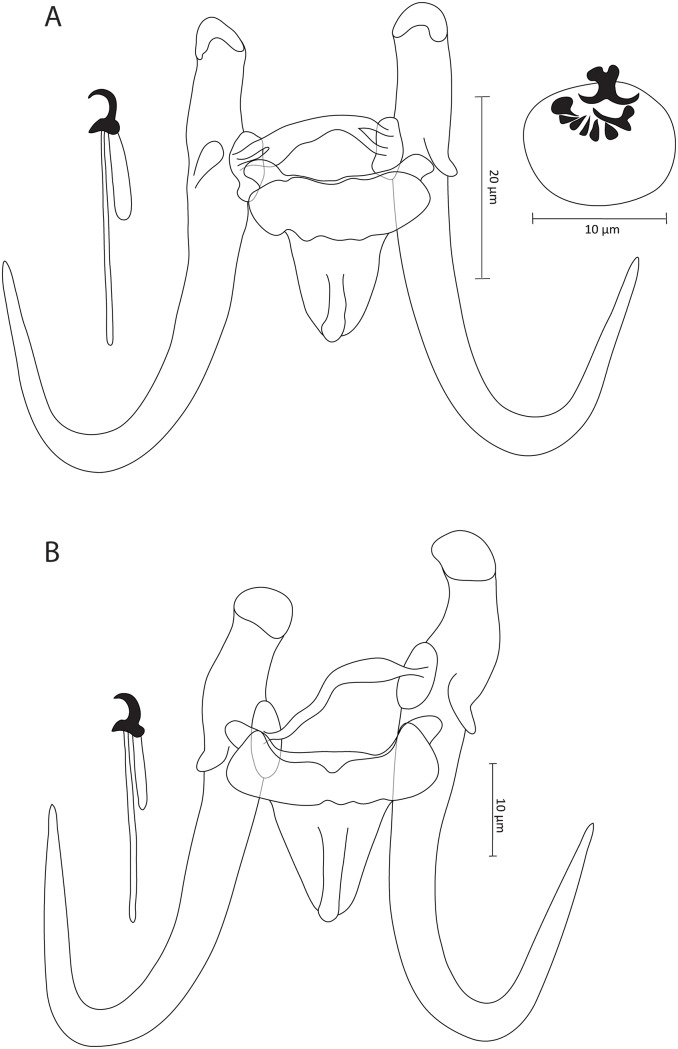
Illustrations of the sclerotized parts of (A) *Gyrodactylus pterocapoetai* sp. nov. from *Pterocapoeta maroccana* Günther, 1902 and (B) *Gyrodactylus qninbai* sp. nov. from *Luciobarbus ksibi* (Boulenger, 1905).

The present study reports the first gyrodactylid species from *P. maroccana* exclusively occurring in Morocco. *Gyrodactylus pterocapoetai* sp. nov. (Figure 4A) is morphologically highly reminiscent of *Gyrodactylus* sp. 6 *sensu* Rahmouni et al. (2023a), described below as *G. shigoleyae* sp. nov. Nevertheless, Rahmouni et al. (2023a) successfully discriminated these 2 species on account of significant genetic divergence (Ziȩtara and Lumme, 2003; Huyse et al., 2004; Rahmouni et al., 2023b). The most relevant morphological feature that makes *G. pterocapoetai* sp. nov. distinguishable from its northwest African counterparts is the posterior bifurcation in the dorsal bar reported for the first time in northwest African *Gyrodactylus* (Rahmouni et al., 2023a).

### Gyrodactylus qninbai sp. nov. (Figure 4B)

**Zoobank registration**: urn:lsid:zoobank.org:act:22336938-6A71-4C47-8926-F003B11CEED3

**Type–host**: *Luciobarbus ksibi* (Boulenger, 1905) (Cyprinidae)

**Type–locality**: Oum Er-Rbia near Chabouka (32°51’32.09” N, 05°37’18.09” W), Morocco

**Additional locality**: Ksob River, Morocco

**Type–material**: 1 holotype and 1 paratype (IPCAS, M–804)

**Site on the host**: Fins

**Infection indices**: 15 specimens studied, *P* = 26.6%, *I* = 1–2 parasite specimens per infected host.

**Etymology**: The specific epithet ‘*qninbai*’ of the type-species honours the ichthyologist Prof. Abdeljebbar Qninba from Mohamed V University (Rabat, Morocco) in recognition of his contribution to fish parasitology research in Morocco.

#### Description

Based on 4 coverslip-flattened specimens. Haptor distinct from the rest of the body. Stout hamuli with well-developed and straight inner roots. Tips have a hat-like cover, with folds present in posterior part of base. Hamuli total length 65 (63.2–67.7), inner root length 18.6 (17.3–19.7), shaft length 48.4 (46.5–50.5) and point length 30 (28.9–31.3). Ventral bar 29 (24.2–32.7) long, 28.3 (26.7–30.2) wide, median 7 (6.3–8.1), with blunt and short bilateral processes that extend outward from bar 3.5 (2.5–6) long, distance between lateral processes 28.6 (27.1–32.3) long, shield-shaped membrane, blunt posteriorly with median ridge, 16.9 (14.2–18.1) long, 16 (14.7–17.5) wide. Dorsal bar straight, with posterior projections near attenuated extremities inserted into terminal plates, 28.8 (27.4–30.8) long, 2.3 (2–2.5) wide at midpoint. Marginal hooks total length 29.3 (27–31), handle length 23.6 (22.5–24.6), filament loop length 11.1 (10.1–12.4), sickle length to shaft attachment 5.8 (5.5–6.6), shaft length of sickle 4.6 (3.9–5.1), sickle distal width 5.1 (4.5–5.5), sickle proximal width 3.7 (3.3–4.1), point length of sickle 1.7 (1.3–2). Sickle foot with conspicuous shelf and globous downward heel. Sickle proper curved distally with straight point positioned above sickle toe. MCO not observed.

#### Remarks

Among the gyrodactylid species studied herein, *G. qninbai* sp. nov. appears morphologically similar to almost all the newly described species, with overlapping measurements of haptoral sclerites. However, *G. qninbai* sp. nov. can be distinguished from *G. agnesei* sp. nov., parasitizing the same cyprinid host, by (i) its slightly shorter hamuli (61–61.4 μm in *G. qninbai* sp. nov. vs. 68.3–71.1 μm in *G. agnesei* sp. nov.), and significantly shorter inner roots (17.1–18.3 μm in *G. qninbai* sp. nov. vs. 23.9–28.5 μm in *G. agnesei* sp. nov.), (ii) the longer bilateral processes of its ventral bar (3.2–6.4 μm in *G. qninbai* sp. nov. vs. 1.8–2.6 μm in *G. agnesei* sp. nov.) and (iii) the absence in *G. qninbai* sp. nov. of the far-reaching point of the marginal hooks sickle observed in *G. agnesei* sp. nov. *Gyrodactylus qninbai* sp. nov. is differentiated from *G. diakini* sp. nov. from *L. rifensis* by its (i) longer bilateral processes of the ventral bar (3.2–6.4 μm in *G. qninbai* sp. nov. vs. 2.1–2.5 μm in *G. diakini* sp. nov.), and (ii) its differently shaped dorsal bar (relatively straight in *G. qninbai* sp. nov. vs. irregular with posterior projections in *G. diakini* sp. nov.). *Gyrodactylus qninbai* sp. nov. also differs from *G. marruecosi* sp. nov. from *L. massaensis* by possessing (i) slightly longer bilateral processes of the ventral bar (3.2–6.4 μm in *G. qninbai* sp. nov. vs. 1.5–2.7 μm in *G. marruecosi* sp. nov.) and (ii) a differently shaped dorsal bar (lacking posterior projections in *G. qninbai* sp. nov., unlike that in *G. marruecosi* sp. nov.).

Shigoley et al. (2023) collected a single *Gyrodactylus* specimen from *L. ksibi* and several from *L. pallaryi*, identifying them as *G. nyingiae*. These specimens were fixed in GAP or Hoyer’s medium, but measurements were only taken from those fixed in Hoyer’s medium, which is known to significantly enlarge sclerotized structures (Fankoua et al., 2017). The *L. ksibi* sample was from the Ksob River in western Morocco, while *L. pallaryi* specimens originated from the Guir River in eastern Morocco. Morphometric data from Shigoley et al. (2023) revealed significant variability in hamuli size within the *L. pallaryi* population. Additionally, their drawings and micrographs showed that *Gyrodactylus* from *L. ksibi* had twisted inner roots, unlike the relatively straight inner roots in *L. pallaryi*. The marginal hooks also differed, with *L. ksibi* specimens exhibiting more pronounced, downward, finger-like toes and curved, thin sickle shafts, compared to the blunt, shelf-less hooks and straight, thicker sickle shafts of *L. pallaryi* specimens.

In our study, we collected *Gyrodactylus* from *L. ksibi* in the Ksob River, fixed in GAP, and found that these specimens resemble those from *L. pallaryi* rather than those from *L. ksibi* reported by Shigoley et al. (2023). Notably, as highlighted by Rahmouni et al. (2023a), the hamuli of Shigoley’s *L. ksibi* specimen closely resemble those of *G. moroccensis* from various northwest African cyprinids, except *L. ksibi*, which hosts *G. pseudomoroccensis*. This suggests that Shigoley et al. (2023) may have misidentified their *L. ksibi* specimen as *G. nyingiae*. Consequently, we describe a new species, *G. qninbai* sp. nov., from *L. ksibi*, distinguished by straight inner roots and different marginal hooks. The absence of detailed dorsal and ventral bar structures in Shigoley et al. (2023) prevents confirmation of their specimen’s identity, which might be *G. moroccensis* or an undescribed species. Additionally, the incomplete ventral bar drawings from *L. pallaryi* highlight the need for redescribing *Gyrodactylus* from this host. Currently, genetic data are unavailable for *Gyrodactylus* specimens from *L. pallaryi* and *L. ksibi*, except for *G. pseudomoroccensis* (Rahmouni et al., 2023a).

### Gyrodactylus shigoleyae sp. nov. (Figure 5A)

**Zoobank registration**: urn:lsid:zoobank.org:act:2519405D-5B00-4C78-BCDE-CCABF5D200C2

**Synonym**: *Gyrodactylus* sp. 6 *sensu* Rahmouni et al. (2023a) (18S: OR807829, ITS: OR807840)

**Type–host**: *Luciobarbus zayanensis* Doadrio, Casal-Lopez and Yahyaoui, 2016 (Cyprinidae)

**Type–locality**: Oum Er-Rbia River near El Borj (33°00”58.07” N, 05°37”48.06” W), Morocco

**Additional locality**: Oum Er-Rbia River at Dar Oul Zidouh (32°18’54.00” N, 06°54’28.07” W), Morocco

**Type–material**: 1 holotype and 1 paratype (IPCAS, M–807)

**Site on the host**: Fins

**Infection indices**: 15 specimens studied, *P* = 50%, *I* = 1–2 parasite specimens per infected host.

**Etymology**: The specific epithet ‘*shigoleyae*’ of the type-species honours the young researcher Miriam Shigoley from Hasselt University (Belgium), who conducted the first description of *Gyrodactylus* from endemic cyprinids of Morocco.

#### Description

Based on 3 coverslip-flattened specimens. Haptor distinct from the rest of body. Stout hamuli with well-developed and slightly curved inward inner roots. Tips have hat-like cover, with folds present in the posterior part of base. Hamuli total length 72.8 (70.5–74.4), inner root length 21.8 (20.5–23.3), shaft length 53.8 (52–55.9) and point length 32.6 (32.6–32.7). Ventral bar 33.1 (29.5–35.9) long, 31.2 (30.7–32.3) wide, median 9.6 (8.6–11.1), with blunt and short bilateral processes that extend outward from bar 4.3 (3.2–4.8) long, distance between lateral processes 32 (31.1–32.5) long, shield-shaped membrane, blunt posteriorly with median ridge, 19.3 (17.6–21) long, 19.5 (17.9–20.9) wide. Dorsal bar straight, possibly with posterior projections near attenuated extremities inserted into terminal plates, 34 (33.2–34.6) long, 3 (2.6–3.2) wide at midpoint. Marginal hooks total length 34.3 (34–34.5), handle length 28.5 (28.4–28.6), filament loop length 12.4 (11.6–12.9), sickle length to shaft attachment 5.7 (5.5–5.8), shaft length of sickle 4.5 (4.4–4.6), sickle distal width 4.7 (4–5.2), sickle proximal width 4.4 (3.7–4.8), point length of sickle 2.2 (2.1–2.3). Sickle foot with conspicuous shelf and rounded heel. Sickle proper with relatively thick shaft that rises forward from the base and curves gradually. MCO oval, 29.3 (29–30.6) in length and 23.9 (22.4–25.4) in width, armed with single apical spine and row of at least 10 spinelets; bilateral pair seems larger.

#### Remarks

**Figure 5.**
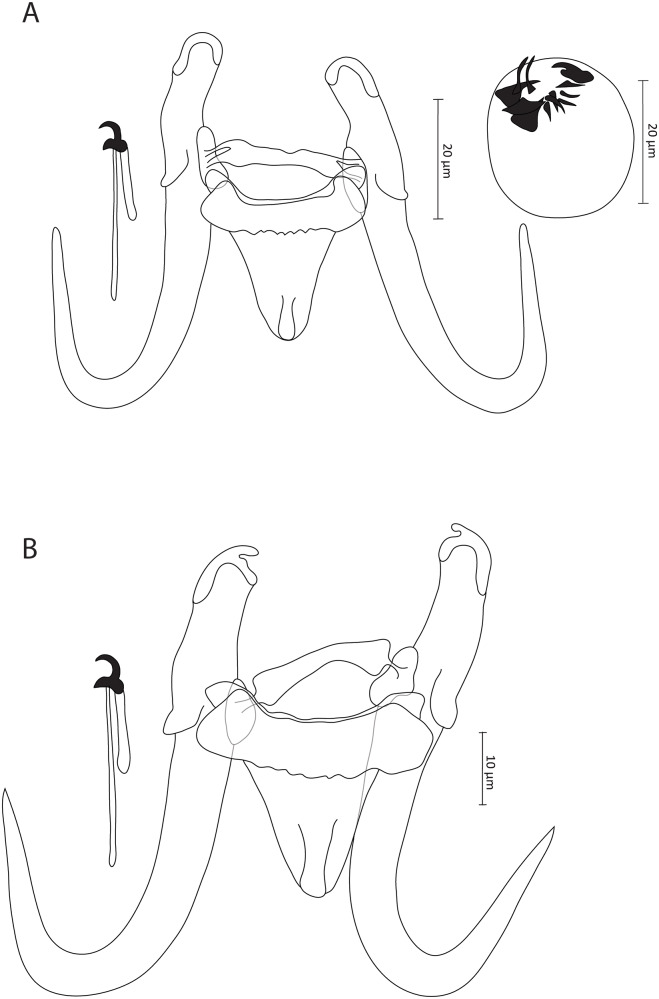
Illustrations of the sclerotized parts of (A) *Gyrodactylus shigoleyae* sp. nov. from *Luciobarbus zayanensis* Doadrio, Casal-lopez and Yahyaoui, 2016 and (B) *Gyrodactylus* sp. 1 ‘*L. massaensis*’ from *Luciobarbus massaensis* (Pellegrin, 1922).

*Gyrodactylus shigoleyae* sp. nov. (Figure 5A) represents the second gyrodactylid species described on *L. zayanensis* from Morocco following *G. moroccensis* (Rahmouni et al., 2023a). Similarly to its congeners, *G. shigoleyae* sp. nov. possesses a distinct haptoral morphology, enabling its differentiation from *G. moroccensis*. While *G. shigoleyae* sp. nov. and its genetically closely related species, *G. pterocapoetai* sp. nov. from *P. maroccana* (Rahmouni et al., 2023a), share a similar haptoral morphotype with bifurcated dorsal bar endings, they can be distinguished from each other by the different sizes of the haptoral sclerites, mainly by (i) the longer hamuli in *G. shigoleyae* sp. nov. (70.5–74.4 μm vs. 58.1–63.1 μm in *G. pterocapoetai* sp. nov.), (ii) the longer ventral bar in *G. shigoleyae* sp. nov. (29.5–35.9 μm vs. 21.9–24.4 μm in *G. pterocapoetai* sp. nov.) and (iii) the longer dorsal bar in *G. shigoleyae* sp. nov. (33.2–34.6 μm vs. 25.7–28.6 μm in *G. pterocapoetai* sp. nov.). Moreover, *G. shigoleyae* sp. nov. and *G. nyingiae* belong to the same morpho-group, regardless of the median ridge feature of the ventral bar, yet they are differentiated by *G. shigoleyae* sp. nov. having (i) a longer ventral bar (29.5–35.9 μm in *G. shigoleyae* sp. nov. vs. 18.6–20.5 μm in *G. nyingiae*), and (ii) a longer membrane (17.6–21 μm in *G. shigoleyae* sp. nov. vs. 12.7–14.5 μm in *G. nyingiae*). It is important to note that the apical spine and the single row of spinelets of the MCO of *G. shigoleyae* sp. nov. that could be visualized were not in their natural position due to the fixation procedure, which likely shifted the garniture (spines) of the MCO.

### Gyrodactylus sp. ‘L. massaensis’ (Figure 5B)

**Type–host**: *Luciobarbus massaensis* (Pellegrin, 1922) (Cyprinidae)

**Number of hosts studied**: 11 specimens

**Type–locality**: Tamrhakht (30°31’33.06” N, 09°38’53.06” W), Morocco

**Site on the host**: Fins

**Infection indices:** 1 specimen found, P = 9.1%.

#### Morphological characterization

Based on a single coverslip-flattened specimens. Haptor distinct from rest of the body. Stout hamuli with well-developed and straight inner roots. Tips may have fleshy hat-like cover, with folds present in posterior part of base. Hamuli total length 72.2, inner root length 26.5, shaft length 54.8 and point length 34.7. Ventral bar 36.4 long, 36.8 wide, median part 10.5, with blunt and short bilateral processes that extend outward from bar, 4.1 long, distance between lateral processes 35.2 long, shield-shaped membrane, blunt posteriorly with median ridge, 19.5 long, 17.6 wide. Dorsal bar slightly curved, with posterior projections near attenuated extremities inserted into terminal plates, 34.6 long, 3.7 wide at midpoint. Marginal hooks total length 35.8, handle length 29.5, filament loop length 13.5, sickle length to shaft attachment 6.3, shaft length of sickle 5.3, sickle distal width 5.2, sickle proximal width 3.6, point length of sickle 2.3. Sickle foot prominent, with conspicuous shelf and rounded downward heel. Sickle proper has relatively thick, short shaft rising forward from the base and curved gradually. MCO not observed.

#### Remarks

This represents the third gyrodactylid species parasitizing northwest African *L. massaensis*, and this study is the second to report a *Gyrodactylus* species on this host after that of Shigoley et al. (2023). Like the latter, insufficient material (*n* = 1) did not allow for a formal description of the species. Since Shigoley et al. (2023) did not provide any meristic data or drawings of the haptoral sclerites, it is hard to determine whether the unidentified specimen we collected is identical to the one they recorded. Nevertheless, we successfully drew the hard parts and made them available for further investigation. The specimen which we collected showed a similar morphotype to all its above-described congeners. Compared to *G. deburonae* sp. nov. from the same host, the single specimen representing *Gyrodactylus* sp. ‘*L. massaensis*’ (Figure 5B) is differentiated by possessing (i) longer hamuli (78.2 μm in *Gyrodactylus* sp. ‘*L. massaensis*’ vs. 46.4–46.8 μm in *G. deburonae* sp. nov.), (ii) a longer ventral bar (36.4 μm in *Gyrodactylus* sp. ‘*L. massaensis*’ vs. 15.5–18.4 μm in *G. deburonae* sp. nov.) with (iii) a longer membrane (19.5 μm in *Gyrodactylus* sp. ‘*L. massaensis*’ vs. 7.9–10 μm in *G. deburonae* sp. nov.), (iv) a longer dorsal bar (34.6 μm in *Gyrodactylus* sp. ‘*L. massaensis*’ vs. 15.9–18.7 μm in *G. deburonae* sp. nov.) and finally (v) longer marginal hooks (35.8 μm in *Gyrodactylus* sp. ‘*L. massaensis*’ vs. 21.9–23.5 μm in *G. deburonae* sp. nov.).

#### Mapping geographical distribution onto parasite phylogeny

**Figure 6.**
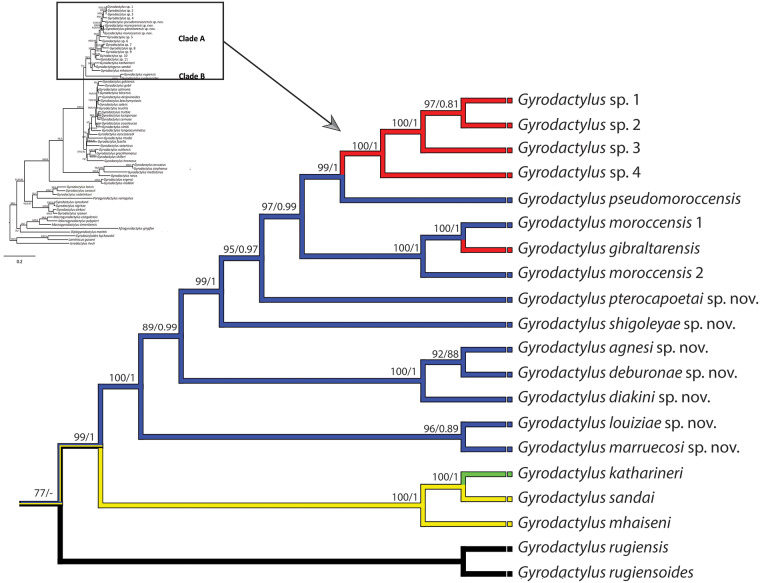
Phylogenetic tree and geographical mapping of the *Gyrodactylus* lineage across the Strait of Gibraltar. The phylogenetic tree presented here was adapted from the one previously generated by Rahmouni et al. (2023a) utilizing Maximum Likelihood and Bayesian methods and included clades A and B only. Geographical regions are indicated as follows: red: Iberia; blue: northwest Africa; yellow: Middle East; green: Europe; black: basal clade B.

The geographical mapping of *Gyrodactylus* species distribution within Clade A (Figure 6) included a total of 18 species, of which 10 species were from northwest Africa, together with *G. mhaiseni, G. sandai* Rahmouni, 2023 from the Middle East, and the European *G. katharineri* Malmberg, 1964. The 2 species basal to clade A formed clade B in Rahmouni et al. (2023a), which clusters *Gyrodactylus* spp. from European gobids. *Gyrodactylus* species from cyprinid fish distributed on each side of the Strait of Gibraltar were polyphyletic, whereas the whole group of *Gyrodactylus* species from cyprinids distributed in northwest Africa and Iberia exhibited a monophyletic origin. Mapping of the geographical distribution onto parasite phylogeny revealed that the *Gyrodactylus* lineage parasitizing northwest African and Iberian cyprinids is of Middle East origin and demonstrated *Gyrodactylus* host-switching from northwest African to Iberian cyprinids, one of these host switching events associated with *Gyrodactylus* diversification in *Luciobarbus* species living in the Iberian Peninsula (*Gyrodactylus* sp. 1–4).

## Discussion

In the present study, we described 9 new *Gyrodactylus* species collected from a range of endemic cyprinids in northwest Africa (Morocco). Most of these species were previously genetically characterized using sequences of the ITS regions, which allowed to reconstruct the phylogeny to reveal their relationships to congeners worldwide (Rahmouni et al., 2023a). In the previous study of Rahmouni et al. (2023a), 3 morphologically specific *Gyrodactylus* species were described from a range of West Mediterranean cyprinids, of which 2 species, namely *G. moroccensis* parasitizing *L. maghrebensis, L. rabatensis, L. rifensis, L. yahyaouii* and *L. zayanensis*, and *G. pseudomoroccensis* from *L. ksibi*, contrasted with a single species, *G. gibraltarensis* found on Iberian *L. graellsii* (Spain). Prior to our study, Rahmouni et al. (2023a) and Shigoley et al. (2023) provided the first morphological data on *Gyrodactylus* specimens collected from a set of endemic cyprinids in Morocco, and successfully described a single species, *G. nyingiae*, from *L. pallaryi* and *L. ksibi*, distributed in the 2 different watersheds in central Morocco. The current study revealing 9 new species brings the total number of *Gyrodactylus* species to 13 in Morocco and to 55 species on the entire African continent. This includes 16 *Gyrodactylus* species limited to cyprinid hosts (Přikrylová et al., 2021, 2024; Shigoley et al., 2023). Even though Shigoley et al. (2023) previously checked for the presence of gyrodactylids on a high number of *Luciobarbus* species in Morocco, as well as on *P. maroccana*, only a relatively low number of parasite specimens were reported, which delayed the formal description of monogenean species. In this study, and to the best of our knowledge, we successfully described the first *Gyrodactylus* species from *C. fritschii, L. massaensis* and *P. maroccana*.

Morphologically, all species described in this study exhibit conservative structures with subtle size variations, accompanied by the characteristic median ridge in the membrane of the ventral bar, a trait previously documented in *Gyrodactylus* species in Eurasia and the West Mediterranean (Pugachev et al., 2010; Rahmouni et al., 2023a). Our previous study Rahmouni et al. (2023a) showed that across the Strait of Gibraltar, several *Gyrodactylus* species have developed a new morphotype during their speciation in the West Mediterranean basins, while also preserving their Eurasian ancestral sclerite features of the haptor. However, the absence of the median ridge in the description of *G. nyingiae*, the first species described from endemic Moroccan cyprinids (Shigoley et al., 2023), may be attributed to oversight or its invisibility during specimen examination.

Among the *Gyrodactylus* species described in our study, *G. benhoussai* sp. nov. was the only species identified on 2 cyprinid species, *L. rabatensis* and *C. fritschii*, sampled from nearby and connected watersheds in central Morocco, which may have facilitated host-switching between cyprinids living in overlapping habitats. Indeed, *Gyrodactylus* species are known for their remarkable ability to switch hosts, thanks to viviparity, which results in the release of fully developed worms actively seeking suitable hosts (Bakke et al., 2007; Boeger et al., 2021). This makes them ideal candidates for investigating historical continental affinities rather than studying inter-host relationships (Boeger et al., 2003). Morphologically, no interspecific variation was observed among specimens of *Gyrodactylus benhoussai* sp. nov. from *L. rabatensis* and *C. fritschii*, with genetic data only available for those from *L. rabatensis*. Further investigations are needed to obtain DNA sequences for *G. benhoussai* sp. nov. associated with *C. fritschii* in order to determine whether there are any genetic differences at host species level. Interestingly, we previously observed genetic divergence in sequences of both the 18S rDNA and ITS regions of *G. moroccensis* from *L. rabatensis* and *L. rifensis*, a result attributed to ongoing speciation (Rahmouni et al., 2023a).

Data on *Gyrodactylus* parasites from *P. maroccana* are provided for the first time in this study, as only a small number of unidentified *Gyrodactylus* specimens were collected in the previous study (Shigoley et al., 2023). *Gyrodactylus pterocapoetai* sp. nov. described from *P. maroccana* and *G. shigoleyae* sp. nov. from *L. zayanensis* both exhibit a similarly bifurcated dorsal bar, consistent with their genetic relatedness (Rahmouni et al., 2023a). Notably, *P. maroccana* naturally inhabits the drainage systems of the Oum Er-Rbia River (type-locality) (Vreven et al., 2016), where our specimens of *L. zayanensis* were collected. Morocco has been colonized in 2 independent freshwater fish waves: the first represented by *P. maroccana*, a potential relic of the Miocene African fauna with a sub-Saharan origin, and the second originating in the Middle East, resulting in a few currently endemic cyprinids like *C. fritschii* (Borkenhagen, 2017). Endemic to the Ksab River system (Froese and Pauly, 2024), *L. zayanensis* may occur in sympatry with *P. maroccana*, potentially favouring the sympatric speciation of gyrodactylids. However, genetic data are still lacking to support this hypothesis. So far, narrow host specificity is observed in *G. pterocapoetai* sp. nov. associated with Northwest African *P. maroccana*, whereas *G. moroccensis*, for instance, parasitizes 5 cyprinid species (Rahmouni et al., 2023a). Sympatric speciation associated with narrow host specificity has been previously demonstrated in *Gyrodactylus*, particularly in Neotropical freshwater fish (Bueno-Silva et al., 2011). Additionally, mitochondrial DNA sequences have revealed that 2 cyprinids – *Labeobarbus bynni occidentalis* (Boulenger, 1911) and *Labeobarbus habereri* (Steindachner, 1912) from the sub-Saharan region – are closely related to *P. maroccana* (Tsigenopoulos et al., 2010; Yang et al., 2015). From a parasitological perspective, it would be intriguing to study *Gyrodactylus* species on the closest relatives of *P. maroccana* to determine the extent of morphological similarities among *Gyrodactylus* parasitizing closely related barbel species across the African continent. In terms of host specificity, Rahmouni et al. (2023a) and the current survey have identified species with 2 different levels of host specificity. As defined by Šimková et al. (2006), *G. moroccensis*, which parasitizes a range of *Luciobarbus* species, and *G. benhoussai* sp. nov. found on *L. rabatensis* and *C. fritschii* can be recognized as intermediate specialists or intermediate generalists, respectively, due to their ability to parasitize congeneric host species or taxonomically and phylogenetically closely related species. Conversely, all other *Gyrodactylus* species described so far from the West Mediterranean, such as *G. pterocapoetai* sp. nov. from northwest Africa and *G. gibraltarensis* from Iberia, are strict specialists, likely able to survive only on a single host species.

This study also describes a new *Gyrodactylus* species, *G. qninbai* sp. nov., collected from *L. ksibi* in the Ksob River, western Morocco. Previously, a single *Gyrodactylus* specimen was reported by Shigoley et al. (2023) from the same host and nearby location, initially identified as *G. nyingiae*, along with specimens from *L. pallaryi* from eastern Morocco. Our detailed morphological analysis of *Gyrodactylus* specimens from both *L. pallaryi and L. ksibi* suggests the potential presence of multiple *Gyrodactylus* species on *L. pallaryi* due to the significant variation in hamuli size. The single specimen from *L. ksibi* mentioned by Shigoley et al. (2023) was likely misidentified as *G. nyingiae*, as its hamuli structure differed notably from those of *L. pallaryi* specimens. Interestingly, the *Gyrodactylus* specimen from *L. ksibi* displayed inner roots with a twisted tip, similarly to *G. moroccensis* found on various *Luciobarbus* species, except *L. ksibi*, which hosts *G. pseudomoroccensis* with straight inner root tips. We cannot conclusively confirm whether the specimen from *L. ksibi* represents *G. moroccensis*, as crucial structural information, such as the dorsal bar, was not provided by Shigoley et al. (2023). However, considering the broad host range of *G. moroccensis* across Moroccan freshwater systems, its occurrence on *L. ksibi* in western Morocco remains plausible. Further investigation is necessary to reassess *Gyrodactylus* parasites on *L. pallaryi* in eastern Moroccan watersheds, and a larger sample size of *L. ksibi* specimens is needed to verify the presence of *G. nyingiae* on this host.

The distributional range of West Mediterranean cyprinid hosts (Froese and Pauly, 2024), their phylogeny (Tsigenopoulos et al., 2003), and the phylogenetic proximity of their *Gyrodactylus* species revealed in our former study (Rahmouni et al., 2023a) as well as the mapping of geographical distribution onto the phylogeny of these *Gyrodactylus* in the present study can be explained by 2 scenarios: the inheritance of gyrodactylid species from common ancestors associated to speciation and host-switching. On the one hand, the former scenario appears plausible given the close phylogenetic relationship among northwest African cyprinids and the high morphological similarity and phylogenetic relatedness observed among their gyrodactylids. Moreover, hybridization is common in freshwater cyprinids, particularly between congeners living in sympatry. For instance, hexaploid Torinae, including *Carasobarbus* Karaman, 1971 and *Pterocapoeta* Gunther, 1902, are hypothesized to have originated from hybridization between Indomalayan tetraploid Torinae and diploid *Cyprinion*-like cyprinids during the westward range extension of the Torinae from the Middle East (Yang et al., 2015; Borkenhagen, 2017). Therefore, our parasitological data provide additional evidence of a shared evolutionary history of cyprinids across continents, particularly in the northwest African region. On the other hand, hybridization between phylogenetically closely related host species may promote host-switching, leading to richer parasite communities, especially concerning monogenean parasites (Šimková et al., 2013; Krasnovyd et al., 2017). Our previous phylogenetic reconstruction (Rahmouni et al., 2023a) and the mapping of the geographical distribution of west Mediterranean *Gyrodactylus* species onto the phylogeny performed in this study revealed 2 host-switches from northwest African to Iberian hosts, one of them associated with specific haptor morphology (Rahmouni et al., 2023a), the other followed by the diversification of *Gyrodactylus* species in *Luciobarbus* of the Iberian Peninsula. This finding implies that Iberian *Gyrodactylus* species may have originated from northwest Africa, and aligns with the geological history of the region; the closure of the Strait of Gibraltar has occurred multiple times due to tectonic movements and changes in sea level, resulting in the formation of a continental bridge connecting Europe and Africa during periods of tectonic uplift or when global sea levels were lower in the late Miocene, 5–6 Ma (Krijgsman et al., 1999). The resulting land corridors allowed biotic interchanges between southern Europe and the northern part of Africa. The current fauna, contrariwise, has been influenced by the reopening of the Strait at the start of the Pliocene, about 5 Ma, which acted as a geographical barrier for gene flow and, thus, vicariance in the Mediterranean lineages (Rosenbaum et al., 2001).

## Conclusions

This study contributes to the current understanding of *Gyrodactylus* diversity in the northwest Africa (Morocco) by formally describing 9 new species parasitizing a range of endemic barbels. These descriptions, combined with previous genetic characterizations, enhance our knowledge of the phylogenetics, distributions and origins of *Gyrodactylus* species. Our findings suggest potential host switches from northwest African to Iberian hosts, supported by the geological history of the region and the intermittent closure of the Strait of Gibraltar. This study also highlights the need for further investigations, particularly regarding genetic differentiation among *Gyrodactylus* species associated with different cyprinid hosts. Future research should focus on obtaining DNA sequences for *Gyrodactylus* species associated with various cyprinid hosts in northwest Africa to better understand their evolutionary relationships and host specificity. Finally, our findings contribute to elucidating the complex evolutionary history and diversification of *Gyrodactylus* lineages in the West Mediterranean, shedding light on the processes driving their distribution and host-parasite associations in this region.

## Data Availability

The data supporting the conclusions of this paper are included within the article. The type-materials of the new species described in this study were deposited in the Helminthological collection of the Institute of Parasitology, Biology Centre of Academy of Sciences of the Czech Republic, České Budějovice (IPCAS) under the accession numbers (M–804-12). The genetic sequence data were already deposited in the GenBank database (see the study by Rahmouni et al. 2023a).
